# Barriers and Facilitators for the Implementation of an Online Clinical Health Community in Addition to Usual Fertility Care: A Cross-Sectional Study

**DOI:** 10.2196/jmir.2098

**Published:** 2013-08-25

**Authors:** Johanna WM Aarts, Marjan J Faber, Anne G den Boogert, Ben J Cohlen, Paul JQ van der Linden, Jan AM Kremer, Willianne LDM Nelen

**Affiliations:** ^1^Radboud University Nijmegen Medical CenterDepartment of Obstetrics and GynecologyRadboud UniversityNijmegenNetherlands; ^2^Radboud University Nijmegen Medical CenterScientific Institute for Quality of HealthcareNijmegenNetherlands; ^3^Isala ClinicsFertility Center IsalaZwolleNetherlands; ^4^Deventer HospitalDepartment of Obstetrics and GynecologyDeventerNetherlands

**Keywords:** community networks, infertility, Internet, quality of health care, patient-centered care

## Abstract

**Background:**

Online health communities are becoming more popular in health care. Patients and professionals can communicate with one another online, patients can find peer support, and professionals can use it as an additional information channel to their patients. However, the implementation of online health communities into daily practice is challenging. These challenges relate to the fact that patients need to be activated to (1) become a member (ie, subscription) and (2) participate actively within the community before any effect can be expected. Therefore, we aimed at answering 2 research questions: (1) what factors are associated with subscription to an online health community, and (2) which are associated with becoming an active participant within an online health community.

**Objective:**

To identify barriers and facilitators as perceived by patients for the implementation of an online health community.

**Methods:**

We performed a cross-sectional study. Three Dutch fertility clinics (2 IVF-licensed) offered their patients a secure online clinical health community through which clinicians can provide online information and patients can ask questions to the medical team or share experiences and find support from peers. We randomly selected and invited 278 men and women suffering from infertility and attending 1 of the participating clinics. Participants filled out a questionnaire about their background characteristics and current use of the online community. Possible barriers and facilitators were divided into 2 parts: (1) those for subscription to the community, and (2) those for active participation in the community. We performed 2 multivariate logistic regression analyses to calculate determinants for both subscription and active participation.

**Results:**

Subscription appeared to be associated with patients’ background characteristics (eg, gender, treatment phase), intervention-related facilitators (odds ratio [OR] 2.45, 95% CI 1.14-5.27), and patient-related barriers (OR 0.20, 95% CI 0.08-0.54), such as not feeling the need for such an online health community. After subscription, determinants for participation consisted of aspects related to participant’s age (OR 0.86, 95% CI 0.76-0.97), length of infertility (OR 1.48, 05% CI 1.09-2.02), and to intervention-related facilitators (OR 5.79, 95% CI 2.40-13.98), such as its reliable character and possibility to interact with the medical team and peers.

**Conclusions:**

Implementing an online health community in addition to usual fertility care should be performed stepwise. At least 2 strategies are needed to increase the proportion of patient subscribers and consequently make them active participants. First, the marketing strategy should contain information tailored to different subgroups of the patient population. Second, for a living online health community, incorporation of interactive elements, as well as frequent news and updates are needed. These results imply that involving patients and their needs into the promotion strategy, community’s design, and implementation are crucial.

## Introduction

In health care today, it is of pivotal importance to take into account the patient’s perspective of care. Patients wish to play an active role, are informed, and prefer involvement in the decision-making process [1-4]. This societal trend is especially visible in the field of reproductive medicine. A plethora of studies have described the importance of involving the patient’s perspective in fertility care and addressed the switch toward more collaboration and partnership with our patients [5-11]. Patients need support from peers, prefer complete and reliable information, wish to communicate online with their clinicians, and want to have easier access to care [12-14]. The developments around Web 2.0, in which the Internet is used as an interactive medium characterized by participation and collaboration between people on the Internet [15-16], provides us with possibilities to fulfill these patients’ needs. Web 2.0 technologies can integrate large amounts of information, which is especially useful in the rapidly evolving field of reproductive medicine in which new insights come and go [17]. Moreover, the Internet can also connect patients to others who are facing the same problem more simply than clinicians can [18-20]. In this respect, the usage of Web 2.0 technologies, such as forums and blogs, are gaining a more prominent position within health care [18,21,22].

The use of these technologies in online health communities in addition to usual care is gaining popularity [18,23]. Previous studies indicated that the integration of Web 2.0 technologies in health care might bring benefits for both patients and professionals in terms of patient empowerment and the possibility to tailor care more appropriately to the needs of patients, also known as patient-centeredness of care [14,21,23-25]. Also, the increasing demand from patients for such communities have led several health care organizations, such as Johns Hopkins Hospital and The Cleveland Clinic, to establish online communities and discussion forums as part of their patient-support services [26]. However, adoption of online health communities is challenging and many interventions lack the ability to maintain usage in the long term [22,27-30]. Potential users should be tempted to join the online health community and, for sustainability, he or she also needs to be challenged to participate actively [30,31]. Chiu and Eysenbach [31] identified 4 stages of using Internet-based interventions that are relevant before positive outcomes can be expected: (1) consideration, (2) initiation, (3) utilization, and (4) outcomes. Every stage has its own barriers, of which adjustment might eventually improve the implementation. Thus, systematically inventorying these factors that facilitate or hinder the use of these interventions is crucial in developing targeted and effective implementation strategies [32].

In this cross-sectional study, we aimed at identifying the barriers and facilitators for the implementation of an online health community in addition to usual fertility care. Therefore, we aimed at answering 2 research questions: (1) what factors are associated with subscription to an online health community, and (2) which are associated with becoming an active participant within an online health community?

## Methods

### Setting

In the Netherlands, couples with impaired fertility can be referred by their general practitioner to a gynecologist in a hospital for further assessment of their fertility problem and for intrauterine insemination (IUI) and ovulation induction (OI) as the first treatment possibilities. In vitro fertilization (IVF), including intracytoplasmatic sperm injection (ICSI), is only performed in 13 IVF-licensed clinics in the Netherlands. In some hospitals without an IVF laboratory, physicians can start up and monitor IVF, perform the oocyte retrieval, and then refer the patient to an IVF clinic for embryo transfer (transport clinic). The Dutch national health care system reimburses the costs of the diagnostic work up, 6 IUI and all OI cycles, and the first 3 IVF cycles. The clinics participating in this study were 2 IVF-licensed clinics and 1 transport clinic.

### Description of an Online Health Community in Addition to Usual Fertility Care

An online health community was constructed as a members-only online community provided by an online platform for online health communities, *MijnZorgnet* (*MyCareNet*) [33]. An online health community offered several functions. First, by means of blogs, professionals could inform their patients about relevant news. Second, it provided 2 separate discussion forums: one in which patients could share experiences and communicate with one another, the other in which patients could ask questions to the medical team. Third, it contained a media gallery in which patients could find digital information leaflets on infertility-related topics. The 3 clinics participating in this study offered such a secured online health community to their own patient population in addition to usual care.

The setup of an online health community was initiated by the head of the department of the 3 different clinics and aimed for improvement of patient-centeredness of care. In every clinic, a nurse or medical assistant was assigned to act as the community manager, responsible for maintenance of the online health community. To become a member, patients used their personal digital identification code to create a profile on the platform of MijnZorgnet [33]. After log-in, patients had to send a membership request to get access. Patients were granted access after subscription with their patient identification number of the hospital. At all 3 clinics, generic information leaflets about the online health community were distributed personally to invite infertile patients to become a member. These patients had their intake visit, underwent a diagnostic work up, or had a fertility treatment, including OI, IUI, or IVF/ICSI.

### Development of Questionnaire

The questionnaire was aimed at identifying aspects relevant to subscribing and active participating in the online health communities. The first part of the questionnaire consisted of questions on background characteristics (eg, age) and characteristics related to their fertility problems (eg, treatment). The second part included items concerning possible barriers and facilitators for subscription to the online health community (part 1), and barriers and facilitators for active participation within the online health community (part 2). Items for this part of the questionnaire were generated from semistructured interviews with 8 patients, conducted for this purpose. All 8 patients had heard about the community, but only 6 decided to subscribe. These patients were asked about the aspects that may impede or facilitate subscription to and participation in the online community and its value for current health care. Interviews were recorded and transcribed verbatim. Transcripts were thematically analyzed by 2 researchers independently and discussed among them to increase coding reliability. Then they divided these items independently into possible barriers and possible facilitators for subscription and participation respectively. They used the 4 domains according the framework of Cabana et al [34] as a framework: patient-related characteristics, intervention-related characteristics, professional-related characteristics, and characteristics of the context in which the intervention was applied. Differences in categorization between researchers were small and consensus was mostly promptly achieved. Although we chose to base the internal consistency of these domains on rigorously performed qualitative analysis, we also calculated Cronbach alpha for each domain as additional information for readers.

These 46 items were converted to a statement. Patients answered at a 4-point Likert scale indicating total disagreement (1) to total agreement (4) with a particular item as a barrier or facilitator for subscribing to or participating in the online health community. All barriers and facilitators were applicable for both subscribing to and participating in the community. Others only applied to active participation, such as “the website doesn’t encourage posting comments or reactions.”

The final questionnaire was pretested among 5 patients resulting in few textual adjustments and the removal of 2 questions.

### Participants and Data Collection

**Figure 1:**
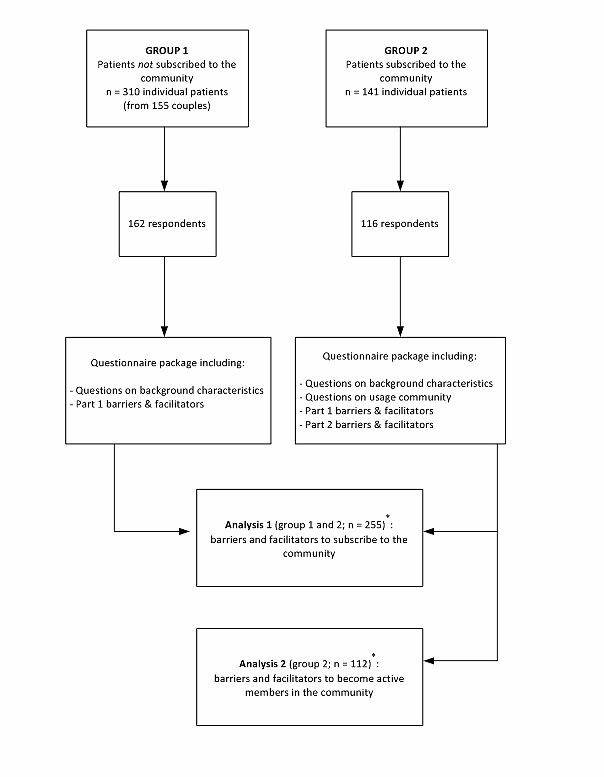
Overview of inclusion procedure participants.

We invited patients who attended 1 of the 3 fertility clinics that participated in this study. We aimed at inviting both patients who were a member of the online health community and patients who were informed about the startup of the online infertility community, but did not subscribe to the community. From the online infertility communities’ members databases, the main researcher randomly selected half of the patients (n=141) to participate in the study. To identify patients who had not subscribed to the online infertility community, the community managers listed all patients that visited the clinic in the previous 2 weeks for an intake consultation, diagnostic assessments, or a fertility treatment. We deleted patients from the lists who already subscribed to the online infertility community. Thereafter, we randomly selected patients from these lists and invited both partners of a couple separately to participate in this study. The proportion of subscribed versus nonsubscribed patients was 1:2, foreseeing a lower response rate of nonsubscribed patients. All participants received a questionnaire package by mail 6 months after the setup of the online infertility community. The questionnaire package was accompanied by instructions, a refusal form, and a stamped return envelope. Participation in the study was voluntary and anonymous. In the Netherlands, institutional ethics committee approval was not required for this study. Participants were sent a reminder at 3 and 5 weeks following the initial mailing, respectively. Figure 1 presents an overview of the data collection and analysis procedure.

### Data Analysis

#### Overview

Data from incoming questionnaires were entered into SPSS version 16.0 for Windows (SPSS Inc, Chicago, IL, USA). Participants who filled out less than 50% of the questionnaire were removed from the database. We used descriptive statistics to present background characteristics of the study population. Answers to open-ended questions were synthesized and categorized. We performed bivariate and multivariate logistic regression analyses to determine factors associated with subscription to (analysis 1) and active participation in (analysis 2) the online infertility community.

#### Independent Variables

In both analyses, we used all patients’ background characteristics (part 1 of questionnaire) combined with the 7 categories of barriers and facilitators (eg, intervention-related category; see Table 1) as independent variables that were based on rigorously performed qualitative analysis. For analysis 1, we used the categories that were composed of those items that were only applicable for subscription (see Table 1). For analysis 2, we used all 7 categories, composed of the 44 single items. Table 1 also shows the statistical reliability of these categories presented as Cronbach alpha. For both analyses, we used per category mean sum scores calculated as the mean score of each individual item divided by the number of items within the category.

#### Dependent Variables

For analysis 1, the dichotomous dependent outcome variable included the question whether they subscribed or did not subscribe to the online infertility community (0=no; 1=yes). In analysis 2, the dependent variable consisted of the activity of a participant within the online infertility community (0 = inactive; 1 = active). We categorized the latter based on self-reported activity. Inactive members had not visited the online infertility community at all after subscription or just a few times without further action. Active users had read the content, visited the online infertility community daily, posted messages, or asked online questions to the medical team. These categories were derived from a social participation ladder [35].

In both analyses, we performed Pearson correlation tests to check for collinearity between the independent variables. Whenever a correlation between 2 variables was more than 0.6, we excluded 1 of those from further analysis. Then, we conducted bivariate logistic regression analysis for each of the independent variables with the 2 different dependent variables. Variables with *P*<.20 were found to be eligible for multivariate regression analysis. A backward selection method was applied, and we considered factors with *P*<.05 significant. We calculated adjusted odds ratios (ORs), *P* values, and 95% confidence intervals (95% CI).

**Table 1:** Barriers and facilitators into domains^a^ resulting from the qualitative analysis.

Barriers and facilitators			Subscription			Active participation	
			Cronbach alpha^b^		Included in the analysis	Cronbach alpha^b^	Included in the analysis
**Barriers**							
	**Related to patient**		.77			.64	
		I’d rather call when I have a question about my treatment		Y			Y
		I’d rather have face-to-face contact with my doctor/nurse		Y			Y
		I don’t need peer support		Y			Y
		I don’t need a website like this		Y			Y
		Participating in this community does not fit my personality		Y			Y
		I have enough knowledge about infertility and treatments		Y			Y
		I have enough people (family and friends) to talk to about my feelings		Y			Y
		I have little Internet experience		Y			Y
	**Related to intervention in general**		.50			.46	
		I didn’t hear about it		Y			Y
		I’m afraid that my privacy is not guaranteed at this website		Y			Y
		I could not find the website and/or community easily		Y			Y
		I experienced problems during log-on with my digital identity					Y
		I don’t know who the other patient members are					Y
	**Related to the intervention’s content**		n/a			.85	
		Too little new information is posted on the website, such as blog messages					Y
		The website does not provide much information (yet)					Y
		The layout of the website doesn’t invite to participate actively					Y
		I think the website is poorly organized					Y
		The website doesn’t encourage posting comments or reactions					Y
		I find using the website difficult/complicated					Y
		The layout of the website consists of too much text					Y
		I have to learn how to use the community					Y
**Facilitators**							
	**Related to the patient**		.52			.54	
		In my daily life I make use of social networking sites, such as LinkedIn or Facebook		Y			Y
		I think it might be fun to use a community like this		Y			Y
		I have few people to talk to about my fertility problems and feelings		Y			Y
		I like to read about new facts (new treatments, research)					Y
		I can help other patients by responding to questions or sharing experiences					Y
	**Related to the intervention**		.75			.83	
		Within the community I can share experiences with peers		Y			Y
		Here I can easily ask questions to my physicians and nurses		Y			Y
		The website has a safe impression because I have to log in using my digital identity		Y			Y
		I can easily find information on this website		Y			Y
		If I forgot to ask something during my appointment, I can do it here afterwards					Y
		Here I can also find information that I wasn’t looking for					Y
		I know that the other members in the community are patients in the same hospital					Y
		I can learn from the questions other people ask					Y
		I can outlet my stories at this website					Y
		The information provided at the website is reliable					Y
	**Related to the context**		.69			.64	
		The virtual infertility community is something new		Y			Y
		My own doctor advised to me to use the virtual infertility community		Y			Y
		The virtual infertility community is a valuable addition to usual care		Y			Y
		Care becomes more patient-centered by offering this community to patients		Y			Y
		Nowadays, everything is digital					
	**Related to the professional**		n/a			.61	
		Also my medical team participates actively within the community					Y
		I like to read the opinion of my doctors about (new) research and treatments					Y
		Because my doctors and nurses answer my questions online, it improves my relationship with them					Y

^a^According the framework of Cabana et al [34].

## Results

### Overview

**Figure 2:**
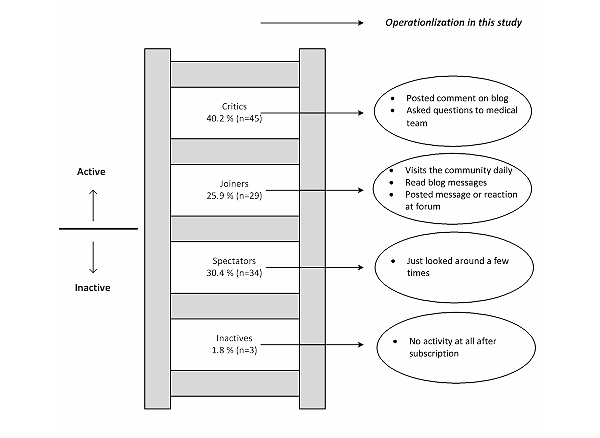
Types of users by self-reported activity according to a participation ladder.

Figure 1 presents a schematic overview of the numbers of patients that were invited, responded, and were eligible for analyses. We invited 141 members from 1 of the 3 clinics’ online health communities to participate in the study and 116 responded (82.3%). In addition, we invited both partners of 155 couples (310 individual patients) among the nonsubscribed population to participate with a response rate of 52.3% (162/310). The main reason for nonparticipation was “not willing to participate in research in general.” In addition, 23 participants were removed from further analyses, because they filled out less than half of the questions on the questionnaire. Table 2 shows the background characteristics of our study population divided into 3 groups: the unsubscribed group of patients, the subscribers, and the active participants. From the total group of participants (N=255), 184 patients had heard about the online infertility community, and 111 had actually subscribed. Figure 2 presents the self-reported activity of the members of 1 of the online health communities (n=112; 1 missing). This number is the sum of the number of participants that we recruited from each of the online health communities that participated in this study.

### Statistical Analyses

Tables 3 and 4 present means of sum scores, including standard deviations, for each subscale. No variables were excluded from the analyses based on collinearity.

#### Bivariate Relationships: Subscribers Versus Nonsubscribers

Table 3 displays the bivariate relationship between each subscale and subscription. All subscales were significantly associated with subscription in these analyses.

#### Bivariate Relationships: Active Versus Nonactive Groups

Table 4 presents the bivariate relationship between each subscale and active participation. All but 2 (ie, barriers related to the intervention in general and the intervention’s content), were significantly associated with active participation.

#### Multivariate Relationships: Subscribers Versus Nonsubscribers

As presented in Table 5, in the multivariate logistic regression analysis, 5 variables predicted the willingness to subscribe to the online health community. for instance, the sum score of the barriers in the patient-related subscale significantly predicted the willingness of patients to subscribe. the higher the sum score, the more patients perceived this category as a barrier. Patients’ characteristics, such as ethnicity, educational level, and average hours of Internet use per week, and context-related and patient-related facilitators did not survive the multivariate regression analysis. the estimation of the explained variance of this multivariate regression model (*R* ^*2*^ =0.48).

#### Multivariate Relationships: Actives Versus Nonactives

As can be seen in Table 6, 3 variables were determinants for the willingness of patients to participate actively within the online health community after subscription. for example, the sum score of intervention-related facilitators was associated significantly with active participation within the online infertility community. Other patients’ characteristics did not survive the multivariate regression analysis (*R* ^*2*^ =0.39).

**Table 2:** Participants’ background characteristics divided in three groups (unsubscribed, subscribed, and participation groups).

Demographic and treatment characteristics		Unsubscribed (n=134)	Subscribed (n=121)	Active (n=74)
**Gender, n (%)**				
	Male	54 (40.6)	12 (9.8)	3 (4.4)
	Female	80 (59.4)	109 (90.2)	71 (95.6)
Age (years), mean (SD)		33.3 (6.1)	33.4 (5.4)	32.2 (3.8)
**Ethnic background,** ^a^ **n (%)**				
	Dutch	124( 93.0)	113 (93.4)	70 (94.1)
	Non-Dutch	10 (7.0)	8 (6.6)	4 (5.9)
**Level of education,** ^b^ **n (%)**				
	Low-middle	62 (46.2)	43 (35.8)	30 (41.2)
	High	72 (53.8)	78 (64.2)	44( 58.8)
Duration of infertility (years), mean (SD)		2.9 (1.9)	3.4 (2.3)	3.8 (2.7)
**Diagnosis, n (%)**				
	Male factor^c^	43 (32.2)	43 (35.7)	27 (36.8)
	Female factor^d^	38 (28.7)	33 (27.7)	21 (27.9)
	Both^e^	19 (14.0)	15 (12.5)	7 (8.8)
	Unexplained	34 (25.2)	27 (22.3)	11 (14.7)
**Treatment type, n (%)**				
	No treatment yet	25 (18.6)	7 (6.0)	2 (3.0)
	ART^f^	58 (43.3)	85( 70.2)	60 (81.0)
	non-ART^g^	50 (37.1)	29 (23.8)	12 (16.0)
**Characteristics related to Internet use**				
	Internet use per week (hours), mean (SD)	17.1 (13.7)	18.9 (13.4)	19.3 (14.1)
	Appreciation community (1-10), mean (SD)	8.2 (1.2)	8.7 (1.0)	9.0 (1.0)

^a^For ethnic background we used the Statistics Bureau Netherlands classification. This Dutch governmental institution classifies ethnicity according to citizens’ country of birth and to that of their parents. Immigrants include both those who are foreign-born (first generation) and those who have at least 1 foreign-born parent (second generation). Categories were: (1) native Dutch, (2) Western or westernized origin (Europe, the United States, Canada, Australia, New Zealand, Japan, and Israel), (3) non-Western origin, immigrants from remaining countries, including Morocco, Surinam, and Turkey.

^b^Low-middle: primary or lower vocational education and secondary or intermediate vocational education; high: higher professional education or university.

^c^Low semen quality.

^d^Irregular ovulation, polycystic ovary syndrome, tubal factor, severe endometriosis, mucus hostility.

^e^Both male and female infertility diagnosis found.

^f^Assisted reproductive technology (ART) encompassed IVF, ICSI, cryopreservation, and testicular sperm extraction.

^g^Non-ART included ovulation induction and intrauterine insemination with or without controlled ovarian stimulation.

**Table 3:** Means (range 1-4), standard deviations, and bivariate relationships of subscribers versus nonsubscribers.

Subscales		Mean sum score^a^ (SD)		Bivariate relationship		
		Subscribers (n=121)	Nonsubscribers (n=134)	OR	95% CI	*P* value
**Barriers**						
	Related to the patient	1.71 (0.43)	1.98 (0.63)	0.40	0.25-0.65	<.001
	Related to the intervention in general	1.41 (0.53)	1.81 (0.76)	0.39	0.26-0.59	<.001
	Related to the intervention’s content^b^	N/A	N/A	N/A		
**Facilitators**						
	Related to the patient	2.19 (0.71)	1.95 (0.74)	1.59	1.12-2.27	.009
	Related to the intervention	2.97 (0.67)	2.60 (0.84)	2.31	1.81-4.85	.001
	Related to the context	2.67 (0.58)	2.40 (0.70)	1.89	1.27-2.83	.002
	Related to the professional^b^	N/A	N/A	N/A		

^a^Mean sum score calculated as the mean score of each individual item divided by the number of items within the category.

^b^N/A: this subscale was only used in analysis of active participation.

**Table 4:** Means (range 1-4), standard deviations, and bivariate relationships of nonactive versus active users.

Subscales		Mean sum score^a^ (SD)		Bivariate relationship		
		Nonactives (n=37)	Actives (n=74)	OR	95% CI	*P* value
**Barriers**						
	Related to the patient	1.92 (0.52)	1.57 (0.52)	0.22	0.08-0.57	.002
	Related to the intervention in general	1.56 (0.57)	1.42 (0.42)	0.47	0.20-1.07	.07
	Related to the intervention’s content	1.71 (0.59)	1.63 (0.59)	0.63	0.33-1.22	.17
**Facilitators**						
	Related to the patient	2.09 (0.65)	2.56 (0.55)	3.12	1.57-6.21	<.001
	Related to the intervention	2.56 (0.67)	3.07 (0.52)	5.32	2.43-11.67	<.001
	Related to the context	2.45 (0.53)	2.81 (0.59)	2.61	1.30-5.26	.007
	Related to the professional	2.51 (0.72)	2.91 (0.68)	2.60	1.42-4.77	.002

^a^Mean sum score calculated as the mean score of each individual item divided by the number of items within the category.

**Table 5:** Multivariate relationship of background characteristics and sum scores of barriers and facilitators to subscribe to the online health community.

Independent variable	OR	95% CI	*P* value	Interpretation
Female	10.52	1.55-71.41	.02	Women more likely to subscribe than men.
IVF treatment	3.18	1.28-7.94	.01	IVF-treated patients more likely to subscribe than non–IVF-treated patients.
Duration of infertility (years)	1.35	1.09-1.69	.007	The longer the patient’s wish for a child, the more likely they will subscribe.
Patient-related barriers	0.20	0.08-0.54	<.001	Patients perceiving patient-related barriers (eg, rather face-to-face) are less willing to subscribe.
Intervention-related facilitators	2.45	1.14-5.27	.02	Patients perceiving intervention-related facilitators are more likely they are to subscribe.

**Table 6:** Multivariate relationship of background characteristics and sum scores of barriers and facilitators to participate actively within the online health community after subscription.

Independent variable	OR	95% CI	*P* value	Interpretation
Age	0.86	0.76-0.97	.02	The younger the patients, the more likely that they will participate.
Duration of infertility (years)	1.48	1.09-2.02	.01	The longer the patient’s wish for a child, the more likely they will participate.
Intervention-related facilitators	5.79	2.40-13.98	<.001	Patients perceiving intervention-related facilitators are more likely they are to participate actively.

## Discussion

### Principal Findings

In this study, we identified barriers and facilitators for subscription and for active participation in an online health community offered in addition to usual fertility care. Subscription appeared to be associated with several patients’ background characteristics, patient-related barriers, and intervention-related facilitators. After subscription, determinants for active participation consisted of participant’s age, length of infertility, and aspects related to characteristics of the online health community itself. to the best of our knowledge, this study is unique because we analyzed the barriers and facilitators for using an Internet intervention into different phases. This provided more detailed information for future implementation strategies, which should take into account these different phases [31].

### Meaning of the Study

This study provides directions on developing a targeted strategy to engage patients, in terms of subscription and active participation, in the online health community as part of the implementation of an online health community [33].

We found that intervention-related characteristics, such as sharing experiences and finding relevant information, facilitated patients’ decisions to subscribe to the online health community and, thus, appealed to most of their needs. However, this did not account for all patients. Our results also show that patient-related barriers are strongly associated with subscription: the more patient-related barriers a patient perceives, the less likely it is that he or she will subscribe. This category consists mostly of internal motivational barriers (eg, no added value) instead of external motivational barriers (eg, lacking correct skills) [36]. It could be the case that a number of people do not feel a fit with their personality. It then could be argued whether we should put too much effort into engaging people who cannot be motivated. However, an implicit explanation of our finding could be based on underlying high anxiety levels, which is not uncommon among infertile patients [37]. Anxious patients generally focus on completing simple tasks of daily living and possibly may not believe that they would benefit from an Internet intervention that comes on top of everything else [38]. However, these patients often have more need for reliable information and support from staff and peers [9], which can be provided by the online community. Therefore, we might need to spend more time identifying patients who might benefit and promoting the community actively among them. in addition, we should evaluate their experiences to optimize the community’s content.

Furthermore, our results show that these patients were primarily female, undergoing IVF treatment, or had a longer duration of childlessness. Based on these results, it may seem clear-cut that we should focus on these groups of patients, but because of the cross-sectional design of our study, it is unknown in what way we should interpret the direction of this association. Either patients meeting these characteristics have more need for an online health community than, for instance, men or patients undergoing non-IVF treatments, or the way in which the content of the online health community is promoted only appeals to this subgroup. for instance, there are gender differences in needs, the experience of infertility, and strategies for coping with fertility-related problems, although infertility is considered a couples’ condition [12,35,39]. Men tend to adopt task-oriented interaction styles [40] and consequently place greater importance on (medical) information than on emotional support groups in contrast to women [41-43]. Furthermore, it is known that patients undergoing diagnostic assessments or a first IUI treatment cycle also have great information needs [44] and suffer from the same emotional impact of being infertile as IVF patients [7,44,45]. Therefore, our results might reflect a lack of acknowledgment of the burden of treatment for men and non-IVF patients, which is still present in infertility services. Thus, the online infertility community could have been unintentionally promoted more prominently among IVF-treated and female patients. in our study, 24% of patients had not heard about the community. This might jeopardize equitability of care, which is also an important component of present-day high-quality care. the Internet has the capability to reach many people at the same time. However, clinics should assess the needs and expectations of different specified target groups within their patient population to tailor the promotion strategy of the online health community more appropriately to these groups. We would generally expect that the process of tailoring would make more content relevant to more people. Clinics should make sure they do not rule out certain subgroups, such as men, in their strategy to promote the community, especially in terms of equitability of care.

In this study, we also investigated those factors that could contribute to active participation within the online health community after subscription. We know from many studies that attrition afterwards is often very high [22,28]. Previous studies have shown that Internet-based interventions only have a fair chance to be effective if members are active participants [29,30]. in our study, almost 70% of subscribers participated actively, which is a fairly high amount. Age and length of infertility were associated with active participation, although these were not strong predictors (given their 95% CIs approaching 1.00). Furthermore, echoing other studies’ results, this study found that intervention-related characteristics play an important role in facilitating active participation in 2 ways. First, the types of technologies used in the community, such as blogs, forums, and wikis, make up the interactive element of the intervention through which patients can share experiences with others and communicate with their doctors. These types of technologies are believed to increase participation and reduce attrition because people get a greater feeling of engagement to the online health community [18,30,46-48]. This is confirmed in our study. Second, the content of the community—a combination of peer-to-peer communication, patient-to-professional communication, and information provision—facilitated active participation, which implies that it fulfilled subscribers’ needs generating value for them. This underlines that it is important to tailor the intervention to patient’s needs.

Although the subscale professional-related facilitators, including active participation from the medical team in the online community, did not survive the multivariate regression analysis, it appeared to have a fairly strong bivariate relationship to active participation. This is in-line with findings in some previous studies: frequent news updates and active participation from clinicians attract patients [47-50]. However, clinicians do also perceive barriers for participating within these types of Internet-based interventions [49,51-54], such as time constraints or lack of knowledge of benefits. Future studies should investigate what specific barriers and facilitators clinicians experience as a next step in the development of a tailored implementation strategy.

### Limitations and Strengths

A strength of our study is that the questionnaire was based on the factors identified by qualitative research. This method assures that the survey is not testing the authors’ personal hypothesis, but represents the complete spectrum of the factors related to adoption of an online infertility community. Another strong point is the fact that we obtained a representative sample of participants and questioned them in a real-life setting instead of an experimental one. the online health community was added to usual care in the clinic they visited. This contributes to the validity of our findings. a difficulty of this study relates to the question whether it can be generalized to other contexts, such as other clinics or other countries. Another context might bring about other barriers and facilitators for the adoption of this intervention. Nevertheless, most factors can be considered universal and probably not specifically related to the Dutch care setting. a second limitation is that we were not able to measure patients’ activity within the online health community objectively, but used self-reported activity instead. Third, it would have been interesting to include every single item from the questionnaire into the regression model. However, our sample size was too small because we needed at least 20 patients for each additional independent variable in the model [55]. Therefore, we narrowed the number of independent variables by using subscales based on rigorously performed qualitative analysis.

### Conclusions

In this questionnaire study, we searched for factors that are associated with subscription to and subsequent active participation in an online fertility community in addition to usual care delivery. We concluded that being female, undergoing IVF treatment, patient-related barriers, and intervention-related facilitators are associated with subscription to the community. Participant’s age, length of infertility, and intervention-related characteristics facilitated the active participation of these subscribers within the online community. These results imply that involving patients and their needs into the promotion strategy, the community’s design, and the implementation plan are crucial.

## Abbreviations

ART: assisted reproductive technology
ICSI: intracytoplasmatic sperm injection
IUI: intrauterine insemination
IVF: in vitro fertilization
OI: ovulation induction
OR: odds ratio
